# Leveraging murine models of the neurofibromatosis type 1 cancer predisposition syndrome to elucidate the cellular circuits that drive pediatric low-grade glioma formation and progression

**DOI:** 10.1093/noajnl/vdae054

**Published:** 2024-06-04

**Authors:** David H Gutmann

**Affiliations:** Department of Neurology, Washington University School of Medicine, St. Louis, Missouri, USA

**Keywords:** ecosystem, low-grade glioma, NF1, neurofibromatosis, optic glioma

## Abstract

Brain tumors are the leading cause of cancer-related death in children, where low-grade gliomas (LGGs) predominate. One common hereditary cause for LGGs involves neurofibromatosis-1 (NF1) gene mutation, as seen in individuals with the NF1 cancer predisposition syndrome. As such, children with NF1 are at increased risk of developing LGGs of the optic pathway, brainstem, cerebellum, and midline brain structures. Using genetically engineered mouse models, studies have revealed both cell-intrinsic (MEK signaling) and stromal dependencies that underlie their formation and growth. Importantly, these dependencies represent vulnerabilities against which targeted agents can be used for preclinical investigation prior to clinical translation.

Children with Neurofibromatosis type 1 (NF1) cancer predisposition syndrome are prone to the development of numerous distinct types of tumors, including low-grade neoplasms of the brain and peripheral nerves. Similar to other autosomal dominant cancer genetic syndromes, individuals with NF1 are born with a germline *NF1* gene mutation in every cell of their body, but only develop tumors when specific cells undergo loss of the remaining normal (functional) *NF1* allele, leading to loss of heterozygosity. Within the central nervous system, optic pathway gliomas (OPGs) predominate, affecting 15% of the NF1 pediatric population. These tumors arise in young children, typically before 7 years of age, and are usually localized to the optic nerves and chiasm (75% of OPGs). While the majority of NF1-OPGs do not cause medical problems, 20%–30% of children with these brain tumors will develop vision loss resulting from degeneration of retinal ganglion cell (RGC)s in the eye or early onset puberty due to hypothalamic involvement. The temporal (age-restricted) and spatial (optic pathway) pattern of tumorigenesis suggests that NF1-OPGs represent developmental abnormalities, reflecting the impact of *NF1* mutation on the normal cellular circuits that orchestrate and maintain brain homeostasis. Moreover, since not all children with NF1 develop OPGs and there is a wide spectrum of symptomatic affectation, defining the cellular and paracrine circuitry that underlies NF1-OPG formation and growth provides unique opportunities to design more targeted therapies and develop prognostic risk stratification tools.

Since surgical biopsy is not part of the standard of care for children with NF1-OPG, many of our insights have derived from the use of *Nf1* genetically engineered mouse models. Prior genetic analyses of human NF1-low-grade gliomas (LGGs) revealed that tumor cells lacking *NF1* gene expression (bi-allelic *NF1* inactivation) are embedded in a microenvironment containing non-cancerous cells harboring only a germline *NF1* gene mutation. In order to parallel the genetics of human NF1-LGG, mice harboring a germline *Nf1* gene mutation in every cell were engineered to undergo somatic *Nf1* loss in a restricted population of progenitor cells during fetal development using conditional transgenesis. Similar to their human counterparts, these *Nf1*-mutant mice develop LGGs within the optic nerve and chiasm.

Employing these instructive preclinical models, several cellular circuits have been identified that each regulates tumor formation and continued growth (Figure 1). First, neuronal activity induced by visual experience (light exposure) triggers a cellular circuit in which *Nf1* mutation results in increased RGC firing in response to light, such that rearing *Nf1*-OPG mice in the dark abrogates glioma initiation.**^1^** This circuit involves *Nf1* mutation- and neuronal activity-dependent elaboration of an enzyme (ADAM10), which releases a membrane-bound growth factor (neuroligin-3; NLGN3) from oligodendrocyte progenitor cells to directly increase tumor cell growth. Second, optic glioma progression in mice also results from the effects of *Nf1* mutation on the basal firing rate of RGCs, leading to activation of an immune cell cascade that supports tumor proliferation.**^2^** In this additional cellular circuit, *Nf1*-mutant neurons release midkine (Mdk) in an activity-dependent manner to stimulate T cells to produce Ccl4, which in turn acts on tumor-associated monocytes (TAMs) to induce the expression of Ccl5, a mitogen critical for *Nf1*-OPG tumor cell growth.**^3^** Third, there is emerging evidence that other circuits exist involving neurotransmitters acting either directly on tumor cells or indirectly through additional cell types.

**Figure 1.**
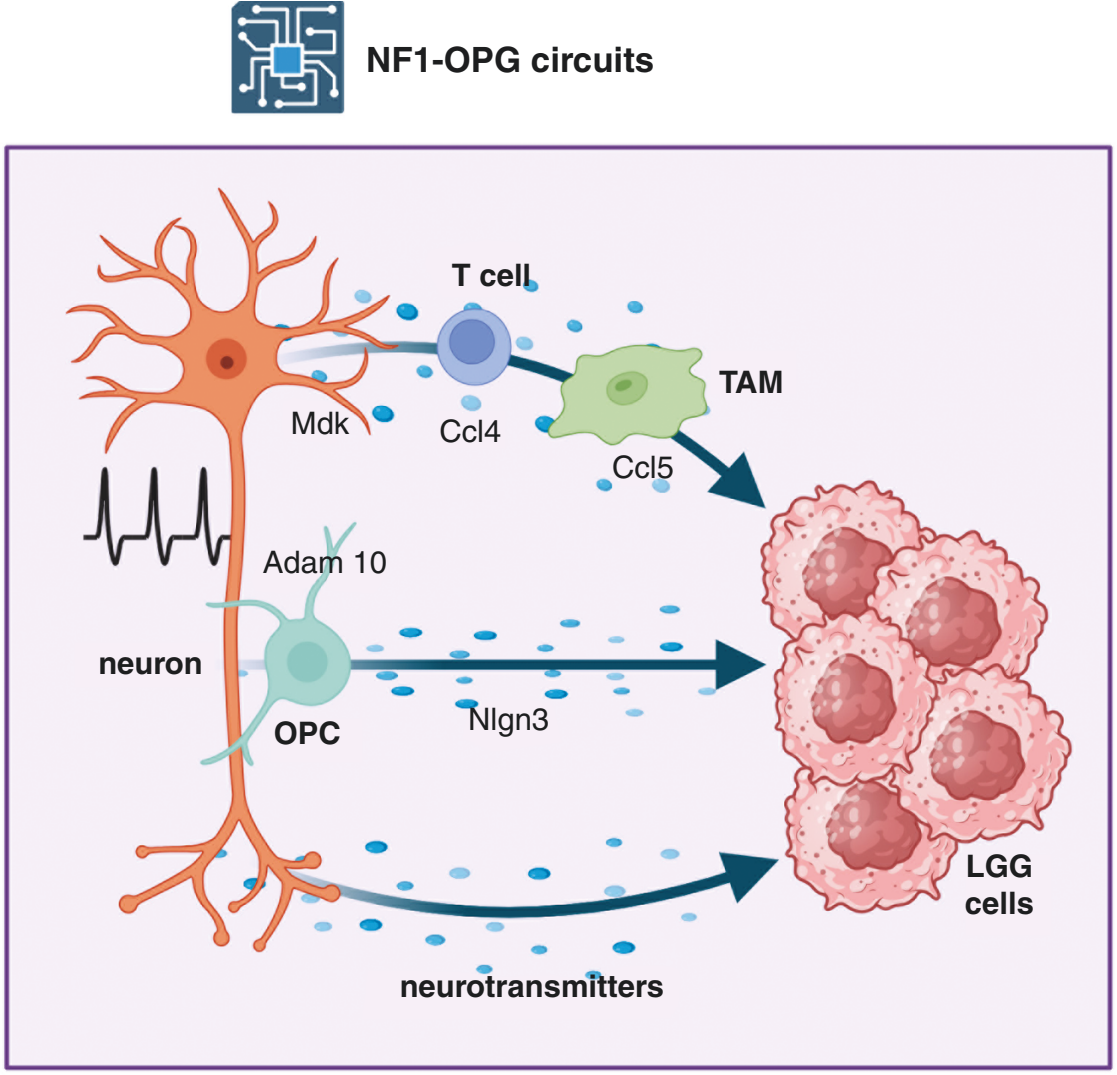
NF1-OPG ecosystems circuits. Several distinct cellular and molecular circuits have been elucidated that control low-grade glioma (LGG; NF1-OPG) initiation and growth. First, *NF1* mutation in neurons leads to increased basal firing rates, such that neuronal activity regulates tumor growth through an immune cell circuit. In this manner, *NF1*-mutant neurons (eg, retinal ganglion cells) produce more midkine in an activity-dependent manner. Midkine acts on T cells to induce Ccl4 expression, which acts on tumor-associated monocytes (TAM) to increase Ccl5 production. Ccl5 is a potent mitogen for NF1-OPG cell growth. Second, *NF1*-mutant neurons in response to light exposure (visual experience) elaborate an enzyme, ADAM10, which cleaves neuroligin-3 (NLGN3) from the surface of oligodendrocyte precursor cells (OPCs). Soluble NLGN3 then binds to a receptor on NF1-OPG tumor cells to drive LGG cell proliferation. Third, *NF1*-mutant neurons elaborate neurotransmitters that bind to their cognate receptors on LGG cells to regulate tumor growth. It is also conceivable that other cellular/paracrine circuits exist that control NF1-OPG initiation and progression.

Elucidating these cellular circuits has already identified numerous unanticipated targets for therapeutic intervention. As illustrated in Figure 2, each cellular element and molecular (paracrine) factor can be inhibited to interrupt specific microenvironmental (stromal) circuits that induce and/or maintain NF1-OPG growth. In this regard, the increased RGC neuronal activity conferred by *Nf1* mutation can be therapeutically exploited by limiting light exposure (or perhaps filtering out specific light wavelengths) and/or normalizing the function of the responsible ion channel (lamotrigine targeting of the HCN channel).**^2^** Similarly, disabling T cell or OPC function with appropriate agents (eg, T cell depletion and ADAM10 inhibitors) abrogates mitogen (Ccl5 and NLGN3) support of glioma growth, respectively. Additionally, blocking the cognate receptors on the tumor cells (NLGN3 receptor and CCL5 receptor) has the potential to eliminate the growth-promoting effects of these stromal mitogens on *Nf1*-OPG expansion. Finally, interrupting neurotransmitter/neurotransmitter receptor signal transduction, either at the level of neurotransmitter bioavailability or receptor function could impair the ability of this paracrine circuit to sustain low-grade glioma growth. Using preclinical *Nf1*-OPG mouse strains, each of the above therapeutic strategies has been evaluated and shown to suppress tumor growth in vivo. Moreover, targeting multiple circuits might yield more effective outcomes by disabling the support networks that maintain tumor homeostasis and progression.

**Figure 2.**
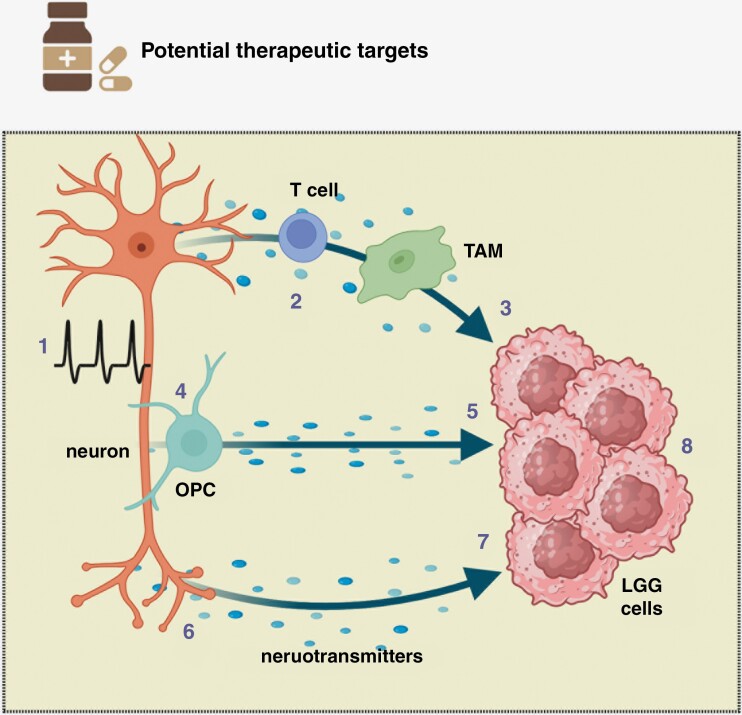
Potential NF1-OPG therapeutic interventions. The existence of several cellular circuits that maintain NF1 low-grade glioma (LGG) cell growth suggests multiple potential treatment opportunities. These include targeting (**1**) neuronal activity through the use of specific ion channel modulators (eg, lamotrigine), (**2**) T cell tumor infiltration and function (eg, immune checkpoint blockade), and (**3**) tumor-associated monocyte (TAM) support of LGG cell growth (eg, colony stimulating factor-1 receptor inhibition). These interventions could operate at the level of the specific paracrine factor or its cognate receptor. Similarly, (**4**) impairing soluble factor release from oligodendrocyte precursor cells (OPC; eg, ADAM10 inhibitors) or (**5**) blocking paracrine factor receptor function could each block tumor growth. In addition, interrupting (**6**) neurotransmitter release and bioavailability or (**7**) neurotransmitter receptor signal transduction might also suppress tumor expansion. All of these stroma-directed therapies could synergize with (**8**) tumor-directed therapies (eg, MEK inhibition) for the treatment of LGG.

Beyond novel therapeutic opportunities, it is likewise possible to envision these circuits as convergence points for risk factors that determine NF1-OPG penetrance and progression (Figure 3). As one example, children with NF1 and asthma have a lower incidence of brain tumors. Using *Nf1*-OPG mice, a cause-and-effect relationship was demonstrated,**^4^** where asthma results in T cell production of decorin, which is important for creating lung pathology. However, when this same paracrine factor is expressed by T cells within the optic nerve tumor, decorin abrogates T cell induction of microglia Ccl5 production and blocks *Nf1*-OPG development. Extending these observations to other systemic exposures, it is conceivable that additional risk factors, including maternal diet, perinatal infection, intestinal microbiota, and nervous system injury, could similarly converge on these circuits to modulate tumor penetrance and growth. Moreover, sex and germline genetics can control tumorigenesis and/or clinical outcome. In this regard, girls with NF1-OPG have higher treatment rates than boys, which reflects sex differences in tumor-induced vision loss. As such, analysis of *Nf1*-OPG mice substantiated this sexually dimorphic effect: female mice have greater RGC loss and nerve fiber layer thinning than their male counterparts.**^5^** Importantly, blocking estrogen function in female *Nf1*-OPG mice attenuated the retinal pathology, suggesting alternative strategies for ameliorating vision loss in children with NF1. Moreover, not all germline *Nf1* gene mutations are functionally equivalent, such that some *Nf1* mutations do not increase RGC neuronal activity or midkine expression, and thus result in no tumors. These observations suggest that specific germline *NF1* gene mutations could establish cancer penetrance set points for children with NF1, which, as predictive biomarkers are discovered and validated, might be used to assess the relative risk of NF1-OPG development in children.

**Figure 3.**
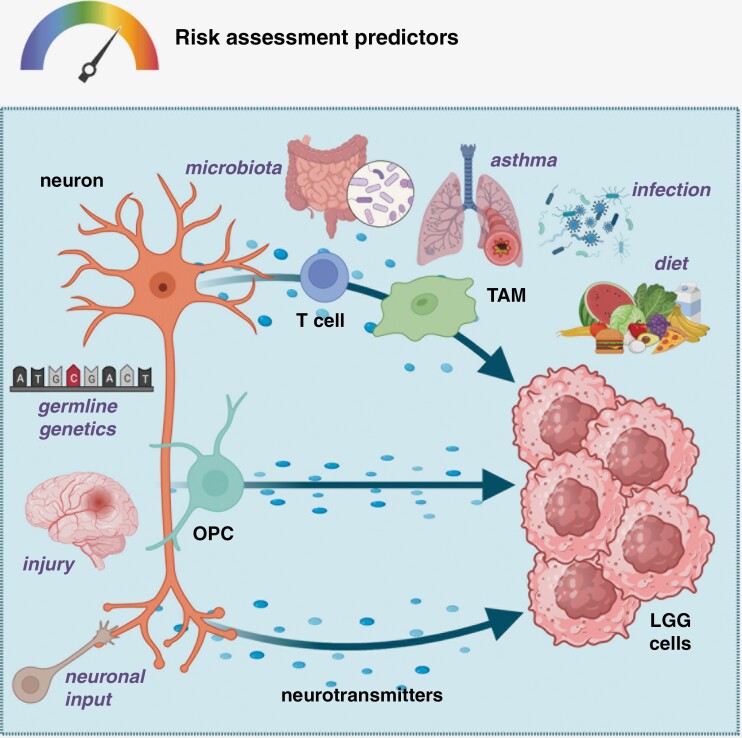
NF1-OPG risk factor predictors. The establishment of cellular circuits that maintain NF1 low-grade glioma (LGG) growth provides likely convergence points for risk factors that modulate tumor formation (penetrance), growth, and response to therapy. In this regard, germline genetics (eg, specific *NF1* gene mutations), brain injury, and other neuronal inputs (eg, visual experience) exert their effects largely at the level of the neuron, leading to changes in immune axis function (T cells and tumor-associated monocytes [TAM], activity-dependent oligodendrocyte precursor cell [OPC]-mediated paracrine factor production; eg, neuroligin-3), and neurotransmitter bioavailability to modify LGG penetrance and growth. In addition, other risk factors, including the intestinal microbiota, asthma, bacterial or viral infection, and diet, could each converge on the immune axis to control LGG formation and maintenance.

Taken together, the use of authenticated *Nf1*-OPG mouse strains has already provided unprecedented insights into the complex systems biology of these pediatric brain tumors relevant to future therapeutic and risk assessment strategies. The addition of multi-omic approaches, including single-cell RNA and ATAC sequencing, proteomics, and metabolomics, will provide greater system biology insights. With these technologies and advances, it might become possible to personalize our approaches to the management and treatment of these low-grade brain tumors in this high-risk population of children. Moreover, the insights provided by NF1 likely extend to sporadic LGGs arising in the general population, as well as the pathobiology of tumors encountered in children with other cancer predisposition syndromes.
